# Association of quantitative sensory testing parameters with clinical outcome in patients with lumbar radiculopathy undergoing microdiscectomy

**DOI:** 10.1002/ejp.1586

**Published:** 2020-06-14

**Authors:** Brigitte Tampin, Helen Slater, Angela Jacques, Christopher R. P. Lind

**Affiliations:** ^1^ Department of Physiotherapy Sir Charles Gairdner Hospital Perth Western Australia Australia; ^2^ Neurosurgical Service of Western Australia Sir Charles Gairdner Hospital Perth Western Australia Australia; ^3^ School of Physiotherapy and Exercise Science Curtin University Perth Western Australia Australia; ^4^ Department of Research Sir Charles Gairdner Hospital Perth Western Australia Australia; ^5^ Institute for Health Research The University of Notre Dame Australia Fremantle Western Australia Australia; ^6^ Medical School University of Western Australia Perth Western Australia Australia

## Abstract

**Background/Aim:**

This study aimed to establish the somatosensory profile of patients with lumbar radiculopathy at pre‐and post‐microdiscectomy and to explore any association between pre‐surgical quantitative sensory test (QST) parameters and post‐surgical clinical outcomes.

**Methods:**

A standardized QST protocol was performed in 53 patients (mean age 38 ± 11 years, 26 females) with unilateral L5/S1 radiculopathy in the main pain area (MPA), affected dermatome and contralateral mirror sites and in age‐ and gender‐,and body site‐matched healthy controls. Repeat measures at 3 months included QST, the Oswestry Disability Index (ODI) and numerous other clinical measures; at 12 months, only clinical measures were repeated. A change <30% on the ODI was defined as ‘no clinically meaningful improvement’.

**Results:**

Patients showed a significant loss of function in their symptomatic leg both in the dermatome (thermal, mechanical, vibration detection *p* < .002), and MPA (thermal, mechanical, vibration detection, mechanical pain threshold, mechanical pain sensitivity *p* < .041) and increased cold sensitivity in the MPA (*p* < .001). Pre‐surgical altered QST parameters improved significantly post‐surgery in the dermatome (*p* < .018) in the symptomatic leg and in the MPA (*p* < .010), except for thermal detection thresholds and cold sensitivity. Clinical outcomes improved at 3 and 12 months (*p* < .001). Seven patients demonstrated <30% change on the ODI at 12 months. Baseline loss of function in mechanical detection in the MPA was associated with <30% change on the ODI at 12 months (OR 2.63, 95% CI 1.09–6.37, *p* = .032).

**Conclusion:**

Microdiscectomy resulted in improvements in affected somatosensory parameters and clinical outcomes. Pre‐surgical mechanical detection thresholds may be predictive of clinical outcome.

**Significance:**

This study documented quantitative sensory testing (QST) profiles in patients with lumbar radiculopathy in their main pain area (MPA) and dermatome pre‐ and post‐microdiscectomy and explored associations between QST parameters and clinical outcome. Lumbar radiculopathy was associated with loss of function in modalities mediated by large and small sensory fibres. Microdiscectomy resulted in significant improvements in loss of function and clinical outcomes in 85% of our cohort. Pre‐surgical mechanical detection thresholds in the MPA may be predictive of clinical outcome.

## 1 INTRODUCTION

Lumbar microdiscectomy is considered a cost‐effective treatment for selected patients with lumbar radiculopathy (Gibson & Waddell, 2007). However, 30 per cent of patients report persistent pain at long‐term follow‐up (den Boer, Oostendorp, Beems, Munneke, Oerlemans, et al., 2006) with associated impacts including reduced work capacity (den Boer, Oostendorp, Beems, Munneke, & Evers, 2006b) and a substantial health economic burden (Parker et al., 2010).

Identification of clinical predictors of outcome in these patients likely involves analysis of many potential interacting factors including psychological and cognitive‐behavioural factors such as fear avoidance behaviour, negative outcome expectancy (den Boer, Oostendorp, Beems, Munneke, & Evers, 2006a; den Boer, Oostendorp, Beems, Munneke, Oerlemans, et al., 2006; Johansson, Linton, Rosenblad, Bergkvist, & Nilsson, 2010), pain catastrophizing (Lautenbacher et al., 2010), depression, anxiety (Chaichana, Mukherjee, Adogwa, Chen, & McGirt, 2011) and physical factors (Rushton, Zoulas, Powell, & Staal, 2018; Werner et al., 2017). While such multidimensional predictors of outcome to inform surgical patient selection are valuable and may guide alternative clinical pathways of care, the predictive value of sensory nerve fibre function has yet to be systematically investigated in people with painful lumbar radiculopathy.

Quantitative sensory testing (QST) is used as a psychophysical test of large and small sensory nerve function enabling the characterization of somatosensory profiles in clinical pain disorders (Maier et al., 2010). Dysfunction typically manifests as sensory loss (i.e. hypoaesthesia) or gain/heightened pain sensitivity to specific stimuli (i.e. hyperalgesia, allodynia) (Hansson, Backonja, & Bouhassira, 2007; Rolke, Magerl, et al., 2006). Gain of function may reflect peripheral or central sensitization, important mechanisms in persistent pain, including neuropathic pain (Jensen & Baron, 2003).

In patients with lumbar radiculopathy, QST has been used to assess the function of sensory nerve fibres in the affected dermatome pre‐ and post‐lumbar decompression surgery (Imoto et al., 2007; Nygaard, Kloster, & Mellgren, 1998; Tschugg et al., 2016). Interpretation of findings is limited, however, as only one study assessed all QST parameters mediated by large and small sensory fibres (Tschugg et al., 2016), and for this study, healthy control data were missing. Furthermore, no study to date has established the QST sensory profile of this patient cohort in relation to their main pain area (MPA), as typically performed in the classification of neuropathic pain (Baron et al., 2017; Haanpää et al., 2011). Sensory profiles in the MPA may differ to profiles in the distal dermatome, as observed in patients with cervical radiculopathy (Tampin, Slater, Hall, Lee, & Briffa, 2012).

Nerve root compression may affect large and/or small sensory fibres (Freynhagen et al., 2008; Nygaard et al., 1998), and the extent of sensory fibre damage may account for heterogeneity of symptoms (Huang et al., 2012). Whether these differences are important as predictors of surgical outcome in radiculopathy has yet to be established. The aims of this study were as follows:

1. To establish the QST somatosensory profile of patients with lumbar radiculopathy/radicular pain pre‐ and post‐microdiscectomy in their MPA and affected dermatome.
2. To investigate if there is an association between pre‐surgical QST parameters and clinical outcome (functional status) post‐surgery.

## 2 MATERIAL AND METHODS

This study was approved by the Sir Charles Gairdner Group Human Research Ethics Committee (HREC 2014‐041). The study participants gave written informed consent to participate and the study protocol adhered to the ethical guidelines of the Declaration of Helsinki. The study was registered with the Australian New Zealand Clinical Trials Registry.

(ACTRN12614001070628: https://www.anzctr.org.au/Trial/Registration/TrialReview.aspx?id=366797).

### 2.1 Sample size

The Oswestry Disability Index (ODI) was used as primary outcome measure to quantify functional status (disability). The scoring of the ODI is between 0–100 (where ‘100’ is considered the maximum disability). Ten points, or a 30% change, is defined as the cut‐off point for qualification of a minimal clinically important change (Raymond W. J. G. Ostelo et al., 2008; Smeets, Koeke, Lin, Ferreira, & Demoulin, 2011). Assuming a 10% rate of persistent pain, a sample size of 40 patients was required to detect 10 units of change from pre‐ to post‐surgery, with a power of 80% and level of significance at 0.05.

### 2.2 Study population

Patients diagnosed with unilateral L5 or S1 radiculopathy due to posterolateral disc prolapse at L4/5 or L5/S1, respectively, were recruited from the elective neurosurgery waitlist (for lumbar microdiscectomy) between October 2014 and April 2016. Seventy‐eight patients gave verbal consent to participate, and of those ultimately 53 patients gave written consent and participated (mean age 38 ± 11 years, 26 females). In order to minimize the variability in outcomes attributed to differences in surgical protocols, patients were selected from one neurosurgeon's waitlist only (CL). The inclusion criteria for the patients were:

- Age 18–65 years;
- Symptom duration of >3 months;
- Clinical diagnosis of lumbar radiculopathy [defined as a conduction block along a spinal nerve or nerve root, manifesting clinically with dermatomal sensory loss or myotomal weakness or reflex changes (Bogduk, 2009)];
- Leg pain in L5 or S1 dermatomal distribution;
- Demonstrable clinically relevant abnormality on imaging studies indicating nerve root compression at L4/5 for L5 radiculopathy and L5/S1 for S1 radiculopathy;
- Listed on the elective neurosurgery surgery waitlist for the procedure of unilateral L4/5 or L5/S1 microdiscectomy.

Exclusion criteria included:

- Diabetes and vascular disease (i.e. any disease affecting the vascular system and potentially affecting the sensation testing in any of the body regions to be assessed, for example Raynaud's disease, peripheral arterial disease);
- Other neurological or psychiatric disease;
- Previous lumbar surgery;
- Insufficient level of English to understand and fill out the questionnaires.

For each patient, an age‐ (±5 years) and gender‐matched healthy control (HC) participant was recruited, with a total of 47 HCs included. Six HCs were assessed on two separate occasions to match their data with two different patients, that is different body regions were tested on each occasion (e.g. L5 lower limb, S1 upper limb). HCs with a history of current pain or a chronic pain condition, or with any of the exclusion criteria described for the symptomatic participant group, were excluded.

### 2.3 Measurement of functional status, pain, quality of life, psychological factors and degree of nerve root compression

Multidimensional aspects of pain were included as clinical measures, consistent with the IMMPACT guidelines (Turk et al., 2006). Functional status was assessed using the ODI (Fairbank & Pynsent, 2000). Based on the ODI score, patients can be classified into five groups: minimal disability (0%–20%), moderate disability (21%–40%), severe disability (41%–60%), crippled (61%–80%), patient is bed‐bound or exaggerates symptoms (81%–100%). The Short Form‐36 health questionnaire (SF‐36v2®) (Ware, 2000) was used to assess health‐related quality of life. The questionnaire contains 36 items measuring health on eight dimensions: physical functioning, role physical, bodily pain, role emotion and mental health plus one time that measures health transition. A physical and a mental composite summary score can be calculated. A higher score indicates better health status.

To determine the severity of symptoms, patients completed numerical rating scales (NRS) (0 = no pain, 10 = maximum pain) for the average pain intensity of back and leg pain in the last 24 hr and over the past week. Bothersomeness of back and leg pain was rated on a 0–5 scale (0 = not at all; 5 = extremely).

The painDETECT questionnaire was used to screen for neuropathic pain and to quantify descriptors of neuropathic pain (Freynhagen, Baron, Gockel, & Tölle, 2006). Each descriptor is weighted from 0 to 5, with ‘0’ indicating the person ‘never feels the sensation’, 1 = ‘the sensation is hardly noticed’, 2 = ‘slightly noticed’, 3 = ‘moderately noticed’, 4 = ‘strongly noticed’ and 5 = ‘very strongly noticed’. A painDETECT score of ≤12 indicates that a neuropathic pain component is ‘unlikely’, a score of ≥19 indicates a likely presence of a neuropathic pain component. Scores between 13 and 18 reflect an ambiguous result.

Inability to work (duration in months/years), confidence in recovery (‘great deal’, ‘moderate’, ‘no confidence’, ‘do not know’) (Ostelo, Vlaeyen, van den Brandt, & de Vet, 2005) and sleep quality over the last week were captured using a 10‐cm Visual Analogue Scale (VAS) with the endpoints 0 cm (good sleep) and 10 cm (bad sleep) (Tampin et al., 2012).

Fear avoidance behaviour was quantified using the Tampa Scale of Kinesiophobia (Swinkels‐Meerwisse, Swinkels, Verbeek, Vlaeyen, & Oostendorp, 2003). This questionnaire consists of 17 items that relate to fear of movement and fear of (re) injury. A score ≥40 is considered to indicate significant kinesiophobia (den Boer, Oostendorp, Beems, Munneke, Oerlemans, et al., 2006).

Pain catastrophizing was measured with the Pain Catastrophizing Scale (PCS) (Sullivan, Bishop, & Pivik, 1995). The PCS contains 13 questions to reflect on past painful experiences and to indicate the degree to which one experiences each of 13 items on thoughts and feelings when experiencing pain. The degree of the experience is indicated on a 5‐point scale from 0 (not at all) to 4 (all the time). A total score of 52 is the maximum achieved. A score greater than 30 indicates a clinically relevant level of catastrophizing (Sullivan et al., 1995).

The Hospital Anxiety and Depression Scale (HADS) was used to screen for the presence of anxiety and depression (Härter, Reuter, Gross‐Hardt, & Bengel, 2001). Two independent scores for anxiety and depression are generated with a maximum score of 21 for each parameter. Scores of ≤10 for each are considered within normal range. The HADS as well as SF36 and the VAS for sleep quality were also completed by HCs.

The degree of nerve root compression was determined by the surgeon based on the grading system documented by Pfirrmann et al. (2004). That is no compromise of the nerve root; the nerve root is in contact with disc material; deviation of the nerve root and nerve root compression.

### 2.4 Quantitative Sensory Testing protocol

**FIGURE 1:**
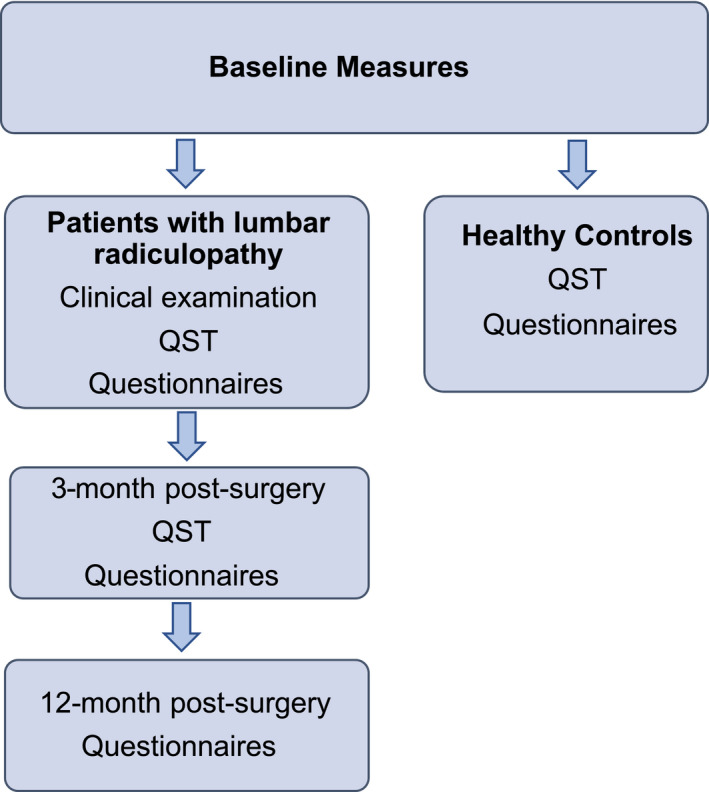
Flow chart of measurements

Baseline assessment was conducted in the week prior to surgery (Figure 1). The initial patient assessment took approximately 2.5 hr. After completion of questionnaires, the patient's pain history, including pain distribution and pain behaviour, symptom duration and intake of medication were recorded. A clinical examination for determination of neurological deficits was then conducted (bedside testing of reflexes, muscle power and sensation of light touch and pin prick).

Standardized QST was performed according to the QST protocol of the German Research Network on Neuropathic Pain (Rolke, Baron, et al., 2006; Rolke, Magerl, et al., 2006). This protocol comprises all of the somatosensory sub‐modalities that are mediated by primary afferents (C‐, Aδ‐, Aβ‐). The protocol included the following assessments: cold and warm detection thresholds (CDT, WDT), the number of paradoxical heat sensations during the procedure of alternating warm and cold stimuli; cold and heat pain thresholds (CPT, HPT); mechanical detection threshold (MDT); mechanical pain threshold (MPT); stimulus‐response functions: mechanical pain sensitivity (MPS) and dynamic mechanical allodynia (DMA); wind‐up ratio (WUR)*;* vibration detection threshold (VDT) and pressure pain threshold (PPT). A detailed description of the methodology can be found in Rolke et al (Rolke, Baron, et al., 2006; Rolke, Magerl, et al., 2006).

QST measurements were taken from the MPA nominated by the patient, as required for the assessment of neuropathic pain (Haanpää et al., 2011), and the contralateral mirror site (Haanpää et al., 2011). Thermal and mechanical detection thresholds were assessed in the relevant dermatome (L5, S1) on the symptomatic side and contralateral mirror site. Testing sites in HC subjects were matched to the sites tested in patients. Patients continued their intake of pain medication throughout the study.

### 2.5 Surgical procedure

All participants undergoing unilateral L4/5 or L5/S1 microdiscectomy were operated on by a single surgeon (CL). After induction of general anaesthesia and endotracheal intubation, patients were positioned prone on a Wilson frame with upper and lower limb joints flexed and with pressure points well padded. Skin on the dorsal aspect of the lumbar region was prepared with alcohol swabs and paramedian placement of a spinal needle performed to plan the midline incision in line with the L4/5 or L5/S1 disc to be operated. Alcoholic chlorhexidine preparation (2%) was applied, followed by iodine‐impregnated incise disposable drapes. With sterile technique, the skin and unilateral thoracolumbar fascia was incised and monopolar diathermy was used to separate unilateral paraspinal muscles from the side of the spinous processes and hemi‐laminae. A dissector was placed in the interlaminar space with lateral x‐ray repeated for confirmation of the level. An operating microscope was brought into the field. A small amount of bone was drilled from the L4 or L5 hemi‐lamina and ligamentum flavum removed to expose the dorsal aspect of the L5 or S1 nerve root in the lateral recess. The nerve root was gently retracted and the sequestered disc prolapse (with or without adjacent loose disc material from the adjacent part of the involved disc space), was removed. Polyglyconate (1 and 2‐0) interrupted sutures were used to close the fascial layers and 3‐0 monofilament absorbable running subcuticular stitch, used to close the skin. Adhesive tapes were placed laterally across the wound and a waterproof dressing applied. The patient was returned to the supine position on the bed then de‐sedated, extubated and recovered before returning to the ward. Most patients were discharged home on postoperative Day one, and all within 3 days of surgery.

### 2.6 Outcome measures 3 and 12 months postoperatively

At 3‐month follow‐up, QST was performed in the patient's previously tested MPA and affected dermatome. Repeat testing of the contralateral side was not performed. QST was not repeated at 12‐month follow‐up for reasons of practicality. Patients had to attend the hospital at 3‐month post‐surgery to be reviewed by the surgeon and QST was performed at the same time. We anticipated that patients would likely not attend the 12‐month appointment, just for QST, as some patients lived quite a distance away from the hospital. The following clinical measures were obtained at 3‐ and 12‐month post‐surgery:

- Functional status (ODI) (Fairbank & Pynsent, 2000)
- Pain intensity (NRS)
- Bothersomeness of back and leg pain, pain descriptors (Freynhagen et al., 2006)
- Change in symptoms (Patient Global Impression of Change Scale) (Dworkin et al., 2005)
- Health‐related quality of life (Ware, 2000)
- Return to work (yes/no)
- Medication intake

### 2.7 Statistical analysis

All data were analysed using the IBM SPSS statistics 24. Age, sleep quality, anxiety and depression scores and the physical and mental component summary scores of the SF‐36 were compared between patients and HCs using parametric (independent *T*‐test) or non‐parametric tests (Mann–Whitney *U*‐Test), as appropriate based on the data distribution. Pre‐ and post‐surgical comparisons for clinical variables for patients with lumbar radiculopathy were assessed using parametric (paired *T*‐test) or non‐parametric tests (Wilcoxon Signed Rank Test), according to the data distribution.

Prior to statistical analysis, QST data were log‐transformed except where raw data were normally distributed (Rolke, Magerl, et al., 2006) and *z*‐transformed for each single parameter using the following expression: *Z*‐score = (Mean_single proband_ − Mean_healthy controls_)/*SD*_healthy controls_ (Rolke, Magerl, et al., 2006). This method allows site‐specific normalization of QST data, where each individual parameter is related to its region and age/gender‐specific reference range, and is displayed as the number of standard deviations above or below the HC mean. *Z*‐values above ‘0’ indicate a gain in function, that is the patient is more sensitive to the tested stimulus compared with healthy controls (hyperalgesia, allodynia), whereas *z*‐scores below ‘0’ indicate a loss of function, referring to a lower sensitivity (hypoalgesia, hypoaesthesia).

*Z*‐values were calculated based on HC data from a database with reference data for the lower limb derived over the period of this study. The database contained reference data from 68 HCs for measurements in the L5 and S1 leg regions including:

- upper leg: L5 lateral thigh; S1 posterior thigh;
- lower leg: L5 lateral side; S1 posterior calf;
- foot: L5 medial foot; S1 lateral foot.

Reference data were obtained from at least eight HC subjects (Blankenburg et al., 2010, 2011) for each MPA nominated by the patients and for various age ranges (20–29 years, 30–39 years, 40–49 years, 50–65 years).

*Z*‐transformation of QST data allowed the documentation of the number of individual participants within the patient group who demonstrated altered QST values (outside 95% confidence interval of reference data), reported as either a loss or gain of function. In contrast to a group comparison, this analysis allows the identification of individual patient differences and the potential identification of subgroups within a specific diagnostic patient group. Frequencies of sensory abnormalities lying outside of the 95% confidence interval (i.e. *z*‐score < −1.96 or >1.96 standard deviation) of our HCs were calculated for each test site on the symptomatic body side.

Additionally, *z*‐transformation of QST data allowed the comparison of sensory profiles between patients who improved at follow‐up post‐surgery (defined as a ≥30% change based on ODI) and those who did not demonstrate clinically important improvement at follow‐up post‐surgery (defined as <30% change on the ODI). Independent *T*‐tests were performed for the comparison of pre‐surgical *z*‐score QST data between these two groups (< or ≥30% change) at 3‐ and 12‐month post‐surgery. In order to assess associations between QST measures and clinical outcome (< or ≥30% change), logistic regression analysis (adjusting for gender, anxiety, depression and pain catastrophizing) was performed for QST parameters that were significantly different between groups.

Fisher's Exact tests for association, including strength of association measures (Phi), was conducted between ODI and PGIC improvement indicators at 3 and 12 months (ODI improvement: ≥30% change on the ODI; no improvement: <30% change on the ODI; PGIC improvement: much improved, very much improved; no improvement: minimally improved to very much worse). Data are presented as mean *SD* unless otherwise indicated. *p*‐values <.05 were considered statistically significant.

## 3 RESULTS

### 3.1 Patient characteristics

The patient characteristics are outlined in Table 1. Symptom duration ranged from four to 50 months, with a mean of 12 months. Twelve patients were diagnosed with L5 radiculopathy and 41 patients with S1 radiculopathy. Twenty‐five patients had the clinical diagnosis of sensory radiculopathy (i.e. only sensory dermatomal deficits, no motor deficits) and the remaining 28 patients had a diagnosis of motor and sensory radiculopathy (sensory and motor deficits). Based on the classification by Pfirrmann et al (Pfirrmann et al., 2004) and as determined by the surgeon (CL), lumbar imaging (MRI, *n* = 40; CT, *n* = 13) demonstrated nerve root compression in 38 patients (72%), nerve root deviation in 13 patients (24%) and for the remaining two patients (4%), the affected nerve root was just in contact with disc material.

**TABLE 1:** Demographics and clinical profiles of patients with lumbar radiculopathy (LxRAD) and healthy control (HC) subjects at baseline (pre‐surgery)

	LxRAD (*n* = 53)	HC (*n* = 47)	*p*‐value
Age (years)^a^ mean/*SD*	38.3 (10.7)	38.3 (10.7)	.997
Gender (female, *n*), *n* (%)	26 (49)	26 (49)	
Symptom duration (months)^a^	11.7 (7.5) (range 4–50)		
Missed days of work, *n* (%)			
0–30 days	23 (43)		
1–2 months	8 (15)		
3–6 months	10 (19)		
6–12 months	6 (11)		
>1 year	3 (6)		
Not applicable (not working)	3 (6)		
Sleep quality during last week (VAS)^b^ (0 = good sleep, 10 = bad sleep)	5.9 (4.0; 0–10) (*n* = 51)	2.3 (3.8; 0–8) (*n* = 45)	<.001^c^
Hospital Anxiety and Depression Scale			
Anxiety score (HADS)^b^	8.0 (6.0; 2–16)	3.0 (5.0; 0–9)	<.001^c^
Within normal range (≤10), *n* (%)	36 (68)	47 (100)	
Depression score (HADS)^b^	6.0 (4.0; 1–16)	0.0 (1.0; 0–3)	<.001^c^
Within normal range (≤10), *n* (%)	47 (89)	47 (100)	
SF‐36			
Physical component^a^	36.8 (6.4) (*n* = 52)	57.2 (2.4) (*n* = 45)	<.001
Mental component^a^	44.0 (10.5) (*n* = 52)	56.1 (3.3) (*n* = 45)	<.001
Pain catastrophizing scale^b^ (>30 clinically relevant level of catastrophizing)	20.0 (18; 1–42)		
Tampa Scale of Kinesiophobia^a^	44.4 (6.9)		

^a^ Data are mean (*SD*).

^b^ Data are median (IQR; min‐max).

^c^ Mann–Whitney *U*‐Test.

For 51 patients, their MPA was located in the affected dermatome in the upper or lower leg, whereas for two patients with S1 radiculopathy their MPA lay within in the L5 dermatome. Nineteen patients indicated their MPA was in the upper leg, whereas 32 patients indicated the lower leg and two patients indicated the foot as their MPA.

Based on the painDETECT questionnaire, the most common pain descriptors felt by patients at a moderate to very strong level were sudden pain attacks (*n* = 42, 79%), numbness (*n* = 31, 58%), tingling (*n* = 29, 55%), burning (*n* = 24, 45%) and slight pressure being painful (*n* = 19, 36%).

### 3.2 Clinical profiles at baseline

The radiculopathy group differed from HC with significantly poorer sleep quality, lower physical and mental component summary scores of the SF‐36 and higher anxiety and depression scores (*p *= <.001 for all comparisons), however 68% of anxiety and 89% of depression scores fell within the normal range (Table 1). The Tampa Scale of Kinesiophobia mean score of 44 (±7) indicated clinically significant fear avoidance levels in the patient cohort. Pain catastrophizing scores for the patient group lay within the normal range (Table 1).

Thirty‐six percent of patients had not worked for more than 3 months due to their pain condition (Table 1). Forty‐two patients (79%) were taking pain medication (Table 2).

**TABLE 2:** Clinical outcome measures of patients with lumbar radiculopathy (LxRAD) taken at baseline, and at 3‐ and 12‐month post‐surgery

	LxRAD (*n* = 53)	LxRAD (*n* = 53)	LxRAD (*n* = 48)	*p*‐value^c^, ^d^	*p*‐value^e^
	Baseline	3 months	12 months		
Oswestry Disability Index^a^ (0–100)	18.1 (6.1)	5.5 (6.5)	5.6 (7.0)	<.001^c^, ^d^	ns
painDETECT^a^ (−1 to 38)	16.1 (1.9)	5.5 (6.0)	5.9 (6.5)	<.001^c^, ^d^	ns
Average leg pain last 24 hr (NRS 0–10)^a^	5.9 (2.0)	1.2 (1.9)	1.5 (2.4)	<.001^c^, ^d^	ns
Average leg pain last week (NRS 0–10)^a^	5.8 (2.0)	1.3 (1.9)	1.6 (2.4)	<.001^c^, ^d^	ns
Bothersomeness leg pain last 2 weeks (0 = no at all, 5 = extremely)	2.7 (0.8)	0.7 (1.0)	0.7 (1.0)	<.001^c^, ^d^	ns
Average back pain last 24 hr (NRS 0–10)^a^	4.7 (2.4)	1.8 (2.4)	2.1 (2.4) (*n* = 47)	<.001^c^, ^d^	ns
Average back pain last week (NRS 0–10)^a^	4.5 (2.5)	2.0 (2.6)	2.0 (2.1) (*n* = 47)	<.001^c^, ^d^	ns
Bothersomeness back pain last two weeks (0 = no at all, 5 = extremely)	2.0 (0.9)	1.1 (1.1)	(1.0) (*n* = 47)	<.001^c^, ^d^	ns
Sleep quality during last week (VAS)^b^ (0 = good sleep, 10 = bad sleep)	5.8 (4.1; 0–10) (*n* = 51)	2.7 (5.4; 0–8.7)	2.5 (5.7; 0–9.3)	.001^c^, ^d^	ns
SF‐36					
Physical component^b^	36.8 (6.4) (*n* = 52)	49.5 (7.6)	49.9 (8.5) (*n* = 46)	<.001^c^, ^d^	ns
Mental component^b^	44.0 (10.5) (*n* = 52)	51.2 (11.0)	53.3 (9.7) (*n* = 46)	<.001^c^, ^d^	ns
Patients with medication, *n*	42 (79%)	15 (28%)	12 (25%)		
Current medication^f^, *n* (%)					
Selective serotonin reuptake inhibitor	2 (4%)	1 (7%)	0		
Serotonin‐norepinephrine reuptake inhibitor	1 (2%)	1 (7%)	1 (8%)		
Tricyclic antidepressant	1 (2%)	0	1 (8%)		
Antiepileptics	17 (32%)	2 (14%)	2 (17%)		
Opioids	18 (34%)	6 (40%)	6 (50%)		
Benzodiazepines	1 (2%)	0	0		
Analgesics	15 (28%)	5 (33%)	6 (50%)		
Non‐steroidal anti‐inflammatories	18 (43%)	4 (26)	5 (42%)		

^a^ Data are mean (*SD*).

^b^ Data are median (IQR).

^c^ Comparison baseline—3‐month post‐surgery.

^d^ Comparison baseline—12‐month post‐surgery.

^e^ Comparison 3 months—12‐month post‐surgery.

^f^ Multiple answers possible.

The mean ODI score of 18 (±6.1) indicated moderate disability for patients with lumbar radiculopathy (Table 2). Eight patients (15%) were categorized as being minimally disabled, 28 patients moderately disabled (53%), 16 patients severely disabled (30%) and one patient classified in the crippled category (2%).

The mean painDETECT score of 16.1 (±1.9) was below the cut‐off established for the classification of neuropathic pain. Sixteen patients were classified by painDETECT as likely having neuropathic pain, 15 patients as not having neuropathic pain and in 22 patients the outcome was unclear (Table 2).

Thirty‐four patients (64%) reported that they had a great deal of confidence that they would recover after surgery, 16 patients (30%) were moderately confident, one patient had no confidence (2%) and two patients (4%) did not know.

### 3.3 QST sensory profiles at baseline

**FIGURE 2:**
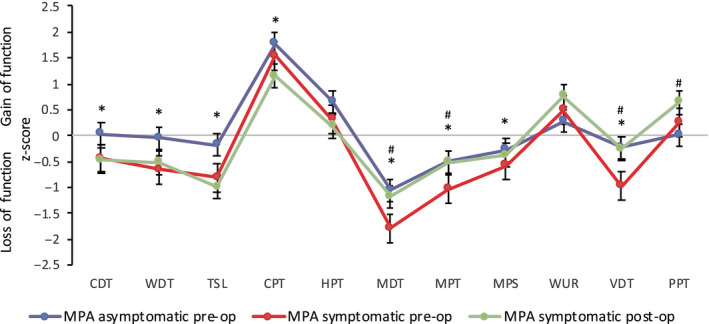
*Z*‐score sensory profiles of the symptomatic (red) and asymptomatic (blue) side pre‐surgery and of the symptomatic side 3‐month post‐surgery (green) in patients with lumbar radiculopathy in the maximal pain area. Error bars indicate the standard error of measurement. CDT, cold detection threshold; WDT, warm detection threshold; TSL, thermal sensory limen; CPT, cold pain threshold; HPT, heat pain threshold; MDT, mechanical detection threshold; MPT, mechanical pain threshold; MPS, mechanical pain sensitivity; WUR, wind‐up ratio; VDT, vibration detection threshold; PPT, pressure pain threshold. *statistically significant difference in the symptomatic leg compared to healthy controls. ^#^statistically significant difference in the symptomatic leg pre‐and post‐surgery

**FIGURE 3:**
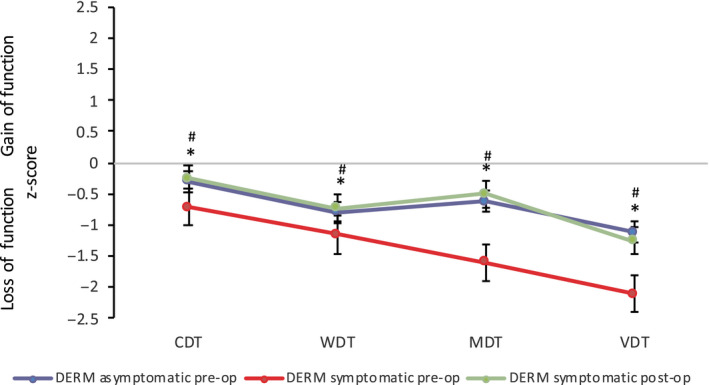
*Z*‐score sensory profiles of the symptomatic (red) and asymptomatic (blue) side pre‐surgery and of the symptomatic side 3‐month post‐surgery (green) in patients with lumbar radiculopathy in the dermatome (DERM). Error bars indicate the standard error of measurement. CDT, cold detection threshold; WDT, warm detection threshold; TSL, thermal sensory limen; CPT, cold pain threshold; HPT, heat pain threshold; MDT, mechanical detection threshold; MPT, mechanical pain threshold; MPS, mechanical pain sensitivity; WUR, wind‐up ratio; VDT, vibration detection threshold; PPT, pressure pain threshold. *statistically significant difference in the symptomatic leg compared to healthy controls. ^#^statistically significant difference in the symptomatic leg pre‐and post‐surgery

For the MPA, the *z*‐score QST sensory profiles of the symptomatic and asymptomatic side are illustrated in Figure 2. Mean values of all QST parameters for both the symptomatic and asymptomatic side fell within the 95% confidence interval of the reference group. For the dermatome, the mean value for VDT in the symptomatic leg fell outside the 95% confidence interval of the reference group, indicating a significantly different value from HCs (Baron et al., 2017). The *z*‐score QST sensory profiles of the dermatome of the symptomatic and asymptomatic side are indicated in Figure 3.

#### 3.3.1 Loss of function

Compared to HCs, patients with lumbar radiculopathy were characterized by a significant loss of function in all sensory fibre populations in the symptomatic leg in both the MPA (thermal, mechanical, vibration detection, mechanical pain threshold, mechanical pain sensitivity *p* < .041) (Figure 2, Table S1) and affected dermatome (thermal, mechanical, vibration detection *p* < .002) (Figure 3, Table S1). There was also a significant loss of function in all sensory fibre populations in the symptomatic leg compared to the asymptomatic leg in both the MPA (thermal, mechanical, vibration detection, mechanical pain threshold, mechanical pain sensitivity *p* < .024) and dermatome (thermal, mechanical, vibration detection *p* < .030) (Table S2). Tables S1 and S2 demonstrate raw QST rather than *z*‐score QST data for easier visual comparison. The frequencies of patients presenting with *z*‐scores indicating a loss of function (< −1.96) were as follows, and shown consecutively for MPA and dermatome: CDT (*n* = 5, 9%; *n* = 6, 12%), WDT (*n* = 4, 8%; *n* = 8, 16%) TSL (*n* = 9, 18%) MDT (*n* = 29, 55%, *n* = 27, 53%) MPT (*n* = 14, 26%), VDT (*n* = 7, 13%; *n* = 15, 29%) and PPT (*n* = 10, 19%).

#### 3.3.2 Gain of function

In comparison with HC data, a gain of function was evident for one nociceptive parameter only, with increased cold pain sensitivity evident in the MPA (*p* = .001) (Figure 2, Table S1). The cold sensitivity in the MPA was, however, not significantly different compared to the patients’ asymptomatic side (Table S2). WUR was present in the MPA in 47 patients. DMA was demonstrated by two patients in the MPA. One patient reported paradoxical heat sensation once in the MPA, two patients reported it twice and one patient reported it three times. One paradoxical heat sensation in response to three cold stimuli is not pathological (Magerl et al., 2010; Rolke, Baron, et al., 2006). The frequencies of patients presenting with *z*‐scores indicating a gain of function (>1.96) in the MPA were as follows: CPT (*n* = 2, 4%), HPT (*n* = 7, 13%), MPS (*n* = 1, 2%), MPT (0%), WUR (*n* = 7, 15%), PPT (*n* = 6, 12%).

### 3.4 Outcomes 3‐month post‐surgery

#### 3.4.1 Clinical outcomes

Three‐month outcome measures were obtained from all 53 patients (100%) (Table 2). Twenty‐three patients (43%) reported that their leg pain had disappeared the day after surgery. One patient developed a spinal abscess at 2‐week post‐surgery, which necessitated multiple hospital visits and >15 days of hospitalization. At a group level, patients had significantly improved at 3 months’ post‐surgery in all clinical outcome measures (*p* < .001) (Table 2). Compared to baseline measures, the frequency of the most commonly reported pain descriptors felt by patients at a moderate to very strong level, was significantly reduced (*p *< .001). Based on the PGIC, 30 patients (57%) reported that they had improved ‘very much’ since surgery, 16 patients (30%) had improved ‘much’ and five persons (9%) had improved ‘minimally’. One person felt ‘minimally worse’ and one person felt ‘much worse’. The latter was not the patient who had developed the spinal abscess. The patient with the abscess reported he had improved minimally at 3‐month post‐surgery. The percentage of patients taking pain medication at 3 months (28%) was reduced compared to pre‐surgery (Table 2).

Thirty‐two patients (60%) had returned to work, whereas 10 patients (19%) had not returned to work. The remaining eleven patients (21%) indicated that this question was not applicable to them (either had not worked pre‐surgery, were studying or were in the process of changing career).

#### 3.4.2 QST sensory profiles 3‐month post‐surgery

At three months post‐surgery, there was a significant improvement in pre‐surgical altered QST measures (mechanical and vibration detection, mechanical pain threshold) in the MPA (*p* < .010), with the exception of thermal thresholds and mechanical and cold pain sensitivity (Figure 2, Table S2). The patient group were more sensitive to pressure pain in their MPA at post‐surgery (*p* = .045). In the dermatome, all pre‐surgical altered QST measurements improved significantly post‐surgery (*p* < .018) (Figure 3, Table S2). One patient demonstrated DMA in the MPA, also evident at baseline. Two patients reported paradoxical heat sensation three times in the MPA. One of these patients had also demonstrated the same response at baseline.

#### 3.4.3 Subgroup of patients with <30% change on ODI

Based on the ODI score, nine patients (17%) documented <30% change on the ODI between baseline and 3‐month post‐surgery. There were no significant differences in baseline clinical measures between patients with <30% change compared to patients with ≥30% change except for leg pain intensity over the last 24 hr (*p* = .046) and bothersomeness of leg pain over the last 2 weeks (*p *< .001) (Table S3). Patients with <30% change had less leg pain [NRS 4.7 (1.9)] pre‐surgery compared to the patients with ≥30% on the ODI [NRS 6.1(1.9)] and the leg pain bothered them less compared to the group with ≥30% change on the ODI (Table S3). Prior to surgery, eight of the nine patients with <30% recovery on the ODI had been identified as having nerve root compression and in one patient the nerve root had been displaced by disc material. Of the patients with <30% change on the ODI, seven did have a great deal of confidence that they would recover after surgery and two had been moderate confident. There was no significant difference in any baseline QST measures in the MPA or dermatome between patients with <30% and with ≥30% change on the ODI. There was a statistically significant association between ODI and PGIC improvement indicators at 3 months (χ^2^(1) = 39.43, *p* < .001), with strength of association measure Phi (φ) = −0.863, *p* < .001) also indicating a strong association between the improvement indicators.

### 3.5 Outcomes 12‐month post‐surgery

#### 3.5.1 Clinical outcomes

Twelve‐month follow‐up data were obtained from 48 patients (91%). Overall, at a group level, patients had significantly improved at 12‐month post‐surgery in all clinical outcome measures (*p* < .001) (Table 2). There was no statistically significant change in clinical outcomes between 3‐ and 12‐month post‐surgery. Compared to baseline measures, the frequency of the most commonly reported pain descriptors felt by patients at a moderate to very strong level was significantly reduced (*p* < .007).

Outcome data on the PGIC were obtained from 47 patients. Based on the PGIC, 30 patients (64%) had improved ‘very much’ since surgery, eight patients (17%) had improved ‘much’ and four persons (8%) had improved ‘minimally’. One person indicated ‘no change’, one person felt ‘minimally worse’ and two persons felt ‘much worse’. In comparison with outcome data at 3 months, the percentage of ‘very much’ improvement had increased by 7%, but the percentage of ‘much’ improvement fell by 13%, one patient had ‘no change’ at all and two patients felt much worse, compared to only one patient at 3 months.

Thirty‐eight patients (79%) had returned to work, three patients (6%) had not returned to work. The remaining seven patients (15%) indicated this question was not applicable to them (three patients had not been in the workforce prior to surgery). In comparison to outcome data at 3 months, the percentage of patients returning to work had increased by 19%.

#### 3.5.2 Subgroup of patients with <30% change on ODI

Seven patients (15%) documented <30% change on the ODI between baseline and 12‐month post‐surgery. Of these seven patients, four also reported <30% change at 3‐month post‐surgery, that is at no follow‐up time point did they show a clinically significant improvement. Out of these four patients, three patients obtained a repeat lumbar MRI post‐surgery which demonstrated granulation tissue around the pre‐surgery affected nerve root and in two cases, swelling of the nerve root. The fourth patient did not undergo any repeat imaging. For the remaining three patients, post‐surgery imaging was obtained for one patient which did not demonstrate any nerve root compromise. Prior to surgery, six of the seven patients had been identified as having nerve root compression and in one patient the nerve root had only contact with disc material. Five patients did have a great deal of confidence that they would recover after surgery and two had been moderately confident. ODI improvement indicators at 12 months were significantly associated with PGIC improvement indicators (χ^2^(1) = 48.00, *p* < .001), with strength of association measure Phi (φ) = −1.000, *p* < .001 also indicating a strong association between the improvement indicators.

There were no significant differences in baseline clinical measures between patients with <30% change and patients with ≥30% on the ODI at 12 months, except for average leg pain intensity over the last week (*p* = .041) (Table S3). Patients with <30% change had less leg pain [4.3 (2.3)] pre‐surgery compared to patients with ≥30% change on the ODI [5.9 (1.8)].

**FIGURE 4:**
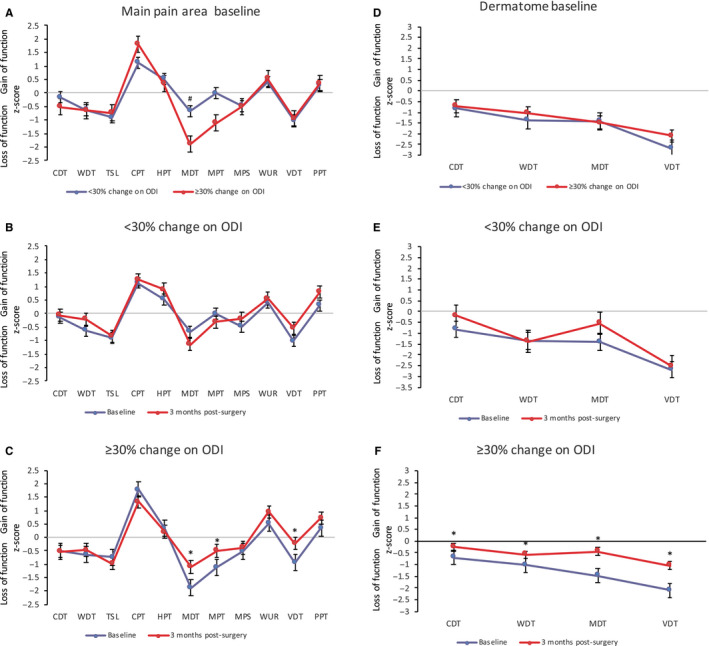
Z‐QST profiles are demonstrated for patients demonstrating <30% and ≥30% change on the Oswestry Disability Index at 12‐month post‐surgery. (A) Baseline *Z*‐score sensory profiles of the main pain area in the symptomatic leg in patients with <30% on the ODI (blue), and ≥30% change on the ODI (red), (B) sensory profiles in the symptomatic leg in patients with <30% change on the ODI pre‐ and 3‐month post‐surgery and (C) in patients with ≥30% change on the ODI pre‐ and 3‐month post‐surgery; (D) Baseline *Z*‐score sensory profiles of the dermatome in the symptomatic leg in patients with <30% change on the ODI (blue) and patients with ≥30% change on the ODI (red), (E) in patients with <30% change on the ODI pre‐ and 3‐month post‐surgery, (F) in patients with ≥30% change on the ODI pre‐ and 3‐month post‐surgery. Error bars indicate the standard error of measurement. CDT, cold detection threshold; WDT, warm detection threshold; TSL, thermal sensory limen; CPT, cold pain threshold; HPT, heat pain threshold; MDT, mechanical detection threshold; MPT, mechanical pain threshold; MPS, mechanical pain sensitivity; WUR, wind‐up ratio; VDT, vibration detection threshold; PPT, pressure pain threshold. *statistically significant difference in the symptomatic leg pre‐and post‐surgery. ^#^statistically significant difference between patients with <30% change on the ODI and patients with ≥30% on the ODI at 12 months

The baseline *z*‐score QST profile in the MPA of patients with <30% change on the ODI showed a significantly smaller loss of function in mechanical detection compared to the *z*‐score QST profile of patients with ≥30% change on the ODI (*p *= .008) (Figure 4a). MDT was significantly associated with group outcome (OR 2.63, 95%CI 1.09–6.37, *p* = .032). The odds of patients with more profound loss of function pre‐surgery improving by at least 30% on the ODI at 12 months were over 2.6 times higher than for patients with less profound loss of function pre‐surgery. The Fisher's exact test indicated no significant difference in the proportion of patients with *z*‐scores < −1.96 *SD* between groups (*p *= .416).Two patients (20%) in the patient group with <30% change on the ODI demonstrated MDT *z*‐scores < −1.96 *SD* compared to 23 patients (56%) in the group with ≥30% change on the ODI.

For patients with <30% change on the ODI, the loss of function in mechanical detection did not improve significantly post‐surgery (Figure 4b), but for patients with ≥30% change on ODI this parameter did improve significantly post‐surgery (*p *< .001), together with measurements of MPT (*p* = .006) and VDT (*p* < .001) (Figure 4c). Patients with <30% change on the ODI demonstrated a trend to increased loss of function in mechanical detection, improved vibration detection and increased pressure sensitivity post‐surgery (Figure 4b).

There were no significant differences in baseline *z*‐score QST profile of the dermatome between patients with <30% and patients with ≥30% change on the ODI at 12 months (Figure 4d). Patients with <30% change on the ODI demonstrated a trend towards a larger loss of function in WDT and VDT compared to patients with ≥30% change (Figure 4d). The baseline loss of function measured in both groups in all parameters (CDT, WDT, MDT, VDT) did not improve significantly for patients with <30% change (Figure 4e). There was a trend to improved function in cold and mechanical detection, but warm and vibration detection remained unchanged (Figure 4e). For patients with ≥30% change in ODI scores baseline loss of function improved post‐surgery for all measurements (*p* < .031) (Figure 4f).

## 4 DISCUSSION

This is the first study to document QST somatosensory profiles in patients with lumbar radiculopathy in their affected MPA and dermatome prior to and post‐microdiscectomy and to explore associations between tested QST parameters and clinical outcome at 3 and 12 months after surgery. The strength of our study lies in the robust methodologies, using clearly defined inclusion criteria for radiculopathy, adhering to best practice recommendations for clinical pain studies (Turk et al., 2006), use of laboratory QST (Baron et al., 2017; Vollert et al., 2016), including small and large fibre function assessment and comparison to matched HC data for each body region assessed. These QST data are also linked with clinical outcomes at 3‐ and 12‐month post‐surgery.

Collectively, our findings indicate in this group of patients with lumbar radiculopathy, a significant loss of function in the symptomatic leg in the MPA (thermal, mechanical, vibration detection, mechanical pain threshold, mechanical pain sensitivity) and affected dermatome (thermal, mechanical, vibration detection) and a gain of function (cold sensitivity) in the MPA at baseline. At 3‐month post‐surgery, this loss of function had improved significantly for measurements of mechanical and vibration detection and mechanical pain threshold in the MPA and for all pre‐surgical altered QST measurements in the affected dermatome. The patient group also became more sensitive to pressure pain in their MPA at 3‐month‐post‐surgery. Baseline loss of function in mechanical detection in the MPA was associated with poorer functional outcome (<30% change on the ODI) at 12 months post‐surgery.

In our patient cohort, a loss of function was the predominant sensory phenotype, and this was evident in the distal dermatome (foot), as well as in the more proximal related dermatomal pain area. These negative sensory signs are indicative of nerve root compromise. Pre‐surgical loss of function occurred for all primary sensory afferents (C‐, Aδ‐, Aβ‐fibres), consistent with findings in some studies on lumbar radicular pain (Andrasinova et al., 2019; Nygaard & Mellgren, 1998; Quraishi, Taherzadeh, McGregor, Hughes, & Anand, 2004; Tschugg et al., 2016). Other studies documented selective loss of function in Aδ‐and Aβ‐fibres (Freynhagen et al., 2008), or dysfunction in only Aδ‐fibres (Mosek, Yarnitsky, Korczyn, & Niv, 2001). However, in these studies not all patients had clinical signs of a radiculopathy.

Nerve fibre damage depends on the compression force and duration (Huang et al., 2012), as well as the degree and speed of compression (Schmid, Coppieters, Ruitenberg, & McLachlan, 2013). While some animal studies showed large myelinated fibres seem more susceptible to nerve compression compared to small fibres (Basbaum, Gautron, Jazat, Mayes, & Guilbaud, 1991; Yoshizawa, Kobayashi, & Morita, 1995), slow compressive nerve compression over 3 months caused preferential degeneration of small fibres (Schmid et al., 2013). Furthermore, abnormal intra‐epidermal nerve fibre density, a measure of small fibre impairment, has been demonstrated in the affected dermatome in 30% of 23 patients with lumbar radiculopathy (Andrasinova et al., 2019). While speculative, the mean symptom duration in our cohort was beyond 3 months (11.7 months), raising the possibility that sub‐clinical small fibre damage may have been present, although parameters still fell within the 1.96 *SD* QST cut‐offs.

Our patient cohort demonstrated only one significant gain of function parameter, namely cold hypersensitivity in their MPA. This finding is consistent with our data from patients with cervical radiculopathy (Tampin et al., 2012), but contrasts to findings in other lumbar radicular pain cohorts, using the same DFNS QST protocol (Andrasinova et al., 2019; Freynhagen et al., 2008; Tschugg et al., 2016, 2018). In fact, in two of these previous studies, no gain of function was detected at all (Andrasinova et al., 2019; Freynhagen et al., 2008), possibly due to QST being performed in the foot dermatome, rather than in the MPA. The underlying mechanisms for cold‐evoked pain likely include peripheral (Serra et al., 2009; Wasner, Schattschneider, Binder, & Baron, 2004), as well as central nervous system mechanisms (Craig, Chen, Bandy, & Reiman, 2000; Jørum, Warncke, & Stubhaug, 2003; Yarnitsky & Ochoa, 1990). While cold hypersensitivity can be a sequel of peripheral nerve injury (Kleggetveit & Jørum, 2010), it has also been evident in patients with no nerve injury (Blumenstiel et al., 2011) and in patients with no pain (Klauenberg et al., 2008). Based on our previous data (Tampin et al., 2012), we anticipated an association between cold hypersensitivity and pain persistency in our cohort, however, cold sensitivity was not associated with poorer clinical outcomes at 3 or 12 months.

While heightened pressure sensitivity has previously been reported in patients with lumbar radiculopathy (Tschugg et al., 2016), in our cohort, no significant difference compared to HC data was demonstrated at baseline. However, as both mixed loss and gain profiles are shown in our pressure pain sensitivity data, both lowered and elevated PPTs may have cancelled each other out in the group analysis. Our cohort showed significantly increased pressure sensitivity at 3‐month post‐surgery, whereas reduced pressure sensitivity at 1 week, with further reduction at 12 months, has been demonstrated by others post‐decompression surgery (Tschugg et al., 2016). The different temporal points in these studies may influence these differing pressure pain sensitivity profiles.

In our cohort, loss of function showed recovery in the dermatome and MPA, except for thermal detection thresholds (small fibres) in the MPA. From the literature, reports on improvement of sensory nerve fibre function post‐surgery in lumbar radiculopathy are conflicting (Nygaard et al., 1998; Zub, Szymczyk, Pokryszko‐Dragan, & Bilińska, 2013), and not readily comparable to our study, due to differing QST methodologies. However, one comparable study reported differential recovery rates: large fibre function recovery in the dermatome as early as 1 week after surgery (Tschugg et al., 2016); small myelinated fibre function improved after 6 months (Tschugg et al., 2016; Zub et al., 2013), whereas C‐fibre function had not improved at 12 months (Tschugg et al., 2016). In comparison, our patient subgroup with <30% change on the ODI at 12 months, demonstrated no significant improvement of C‐fibre and large fibre (vibration detection) function in the dermatome at 3‐month post‐surgery, and a further loss of large fibre function (mechanical detection) in the MPA. It remains unknown if these sensory alterations persisted at 12‐month post‐surgery, as we did not repeat QST testing. Reasons for these divergent outcomes remain speculative. Pre‐surgical loss of function may have been caused by ischaemic conduction block due to the nerve root compression, which improved with normalization of blood flow to the nerve root post‐surgery (Hida, Naito, & Kubo, 2003). This may explain patients reporting immediate post‐surgery pain relief (Tschugg et al., 2016), as also observed in 43% of our patients. Regeneration of demyelinated fibres with preserved axons and associated functional recovery would likely occur faster than regeneration of more specific axonal damage. In fact, it is not known if in the above studies, including ours, nerve root compression caused any structural changes of the tested sensory fibres. Whereas QST provides information about the function of somatosensory afferents, it does not provide information about structural nerve fibre integrity.

Interestingly, sensory profiles differed between the dermatome and MPA, that is thermal thresholds improved in the dermatome, but not significantly in the MPA. Furthermore, loss of mechanical detection in the MPA was associated with clinical outcome at 12 months, but not for MDT in the dermatome. These observations may point to differing underlying pathophysiological mechanisms in radiculopathy. While loss of function in a pain‐free dermatome may reflect structural nerve fibre damage, loss of function in a painful area may reflect secondary hypoesthesia mediated at the spinal cord level (Geber et al., 2008; Magerl, Wilk, & Treede, 1998) or possibly cortical changes associated with central plasticity (Geber et al., 2008; Westermann et al., 2011). Our findings suggest that the assessment of sensory profiles in the MPA is important in the search for associations with pain persistency post‐microdiscetomy.

Surprisingly, for the majority of our patients, the baseline anxiety, depression and pain catastrophizing scores were within normal range. However, this is not necessarily unheard‐of. In a large cohort of patients with painful radiculopathy (*n* = 2094), only 4.6% of patients had severe anxiety, 4.8% had severe depression and 37% had moderate depression (Mahn et al., 2011). It is possible that our patients might have developed better coping strategies for their pain conditions compared to other cohorts. One reason may be that our patient group had a specific reason for their pain with a clear condition‐specific diagnosis provided and they were given the prospective of improvement with surgery. In contrast, patients with, for example, chronic low back pain often do not know the cause of their pain, as documented by Daniel et al. (2008) comparing psychological and physical function in patients with post‐herpetic neuralgia and patients with nociceptive low back pain. Factors are likely complex and may represent a case mix too.

## 5 LIMITATIONS OF THE STUDY

We chose the conservative cut‐off of <30% change on the ODI (i.e. 10 points) as a clinically meaningful difference, others suggest a cut‐off of 20 points to ensure meaningful change after surgery (Solberg, Johnsen, Nygaard, & Grotle, 2013). This suggested cut‐off was not feasible as 36 (68%) of our patients scored <20 points at baseline (i.e. creating a potential floor effect). However, further analysis revealed that our results were comparable using the PGIC, which as an outcome measure, is independent of baseline values. Our subgroups of patients with <30% change on the ODI at 3 and 12 months were small and the risk of a type II error cannot be excluded. The intake of pain medication may have influenced QST and clinical outcomes. Surgery was performed by the same surgeon which limits generalizability of our results.

In conclusion, our patients’ predominant sensory phenotype was ‘loss of function’ in the pain‐free foot dermatome, as well as in the painful area, likely reflecting nerve root compromise. Microdiscectomy resulted in significant improvements in the affected somatosensory parameters and improved clinical outcomes in 85% of our cohort. Pre‐surgical MDTs measured in the MPA are associated with clinical outcome, as measured in our study, however larger high‐quality trials are required to confirm these findings.

## CONFLICT OF INTEREST

All authors declare no conflicts of interest.

## AUTHORS' CONTRIBUTIONS

BT, HS and CL conceptualized the experiment and acquired funding. BT and CL collected data. All authors were involved in data analysis and discussed the results. BT drafted the first version of the manuscript. All authors provided input to the final version of the manuscript.

## Supporting information

Table S1

Table S2

Table S3
