# Supplementary material for: Association of quantitative sensory testing parameters with clinical outcome in patients with lumbar radiculopathy undergoing microdiscectomy
Source: Eur J Pain. 2020 Jun 14;24(7):1377–92. doi: 10.1002/ejp.1586 (PMC7496563; doi:10.1002/ejp.1586)
Supplement: Supplementary file 1 — Table S1 [file EJP-24-1377-s001.doc]

**Supplementary Table 1**

QST parameters in healthy controls and patients with lumbar radiculopathy (LxRAD)

in the main pain area and dermatome. Data are shown as mean for untransformed data

(CPT, HPT, VDT) ± standard deviation and retransformed means.

|  | MAIN PAIN AREA | | |
| --- | --- | --- | --- |
| Parameters | Healthy controls | LxRAD | *P** |
| CDT (°C) | 2.6 | 3.6 | **0.019** |
| WDT (°C) | 4.8 | 6.1 | **0.040** |
| TSL (°C) | 8.0 | 10.3 | **0.003** |
| CPT (°C) | 8.9±6.8 | 14.9±8.7 | **0.001** |
| HPT (°C) | 47.3±2.7 | 47.0±2.7 | 0.300 |
| MDT (mN) | 1.3 | 9.9 | **0.000** |
| MPT (mN) | 33.6 | 75.3 | **0.000** |
| MPS (NRS_100_) | 0.6 | 0.4 | **0.000** |
| WUR (ratio) | 2.2 | 2.3 | 0.173 |
| VDT (x/8) | 5.5±1.3 | 4.3±1.4 | **0.000** |
| PPT (kPa) | 382 | 352 | 0.651 |
|  | DERMATOME | | |
|  | Healthy controls | LxRAD | *p* |
| CDT (°C) | 4.1 | 6.1 | **0.001** |
| WDT (°C) | 5.6 | 7.6 | **0.001** |
| MDT (mN) | 1.6 | 11.0 | **0.000** |
| VDT (x/8) | 6.8±0.9 | 5.6±1.5 | **0.000** |

CDT: cold detection threshold; WDT: warm detection threshold; TSL: thermal sensory limen; CPT: cold pain threshold; HPT: heat pain threshold; MDT: mechanical detection threshold; MPT: mechanical pain threshold; MPS: mechanical pain sensitivity; WUR: wind-up ratio; VDT: vibration detection threshold; PPT: pressure pain threshold. *p-values are given for statistical analysis using z-score QST data; bold numbers indicate statistically significant difference
