# Supplementary material for: Association of quantitative sensory testing parameters with clinical outcome in patients with lumbar radiculopathy undergoing microdiscectomy
Source: Eur J Pain. 2020 Jun 14;24(7):1377–92. doi: 10.1002/ejp.1586 (PMC7496563; doi:10.1002/ejp.1586)
Supplement: Supplementary file 2 — Table S2 [file EJP-24-1377-s002.doc]

**Supplementary Table 2**

Comparisons of QST parameters in patients with lumbar radiculopathy in the main pain area (MPA) and dermatome on the

symptomatic side and matched asymptomatic side at baseline (pre-surgery) and comparison of QST parameters

on the symptomatic side pre- and three months post-surgery. Data are shown as mean for untransformed

data (CPT, HPT, VDT) ± standard deviation and retransformed mean

|  | MPA baseline pre-surgery | | |  | MPA post-surgery | |
| --- | --- | --- | --- | --- | --- | --- |
| Parameters | Asymptomatic | Symptomatic | *p*^a^ |  | Symptomatic | *p*^b^ |
| CDT (°C) | 2.8 | 3.6 | **0.002** |  | 3.4 | 0.882 |
| WDT (°C) | 4.7 | 6.1 | **0.003** |  | 5.7 | 0.478 |
| TSL (°C) | 8.5 | 10.3 | **0.003** |  | 10.7 | 0.467 |
| CPT (°C) | 14.2±8.4 | 14.9±8.7 | 0.626 |  | 12.4±8.8 | 0.413 |
| HPT (°C) | 46.5±2.9 | 47.0±2.7 | 0.063 |  | 47.6±3.2 | 0.582 |
| MDT (mN) | 4.2 | 9.9 | **0.000** |  | 4.8 | **0.001** |
| MPT (mN) | 44.9 | 75.3 | **0.001** |  | 48.1 | **0.009** |
| MPS (NRS_100_) | 0.5 | 0.4 | **0.023** |  | 0.4 | 0.166 |
| WUR (ratio) | 2.3 | 2.3 | 0.789 |  | 2.5 | 0.383 |
| VDT (x/8) | 5.1±1.4 | 4.3±1.4 | **0.000** |  | 5.1±1.5 | **0.000** |
| PPT (kPa) | 371 | 352 | 0.296 |  | 294 | **0.045** |
|  | Dermatome baseline pre-surgery | |  | Dermatome post-surgery | | |
|  | Asymptomatic | Symptomatic | *p*^a^ |  | Symptomatic | *p*^b^ |
| CDT (°C) | 4.5 | 6.1 | **0.001** |  | 4.5 | **0.001** |
| WDT (°C) | 6.6 | 7.6 | **0.029** |  | 6.5 | **0.017** |
| MDT (mN) | 4.1 | 11.0 | **0.000** |  | 3.5 | **0.000** |
| VDT (x/8) | 6.1±1.4 | 5.6±1.5 | **0.000** |  | 6.0±1.5 | **0.005** |

CDT: cold detection threshold; WDT: warm detection threshold; TSL: thermal sensory limen; CPT: cold pain threshold;

HPT: heat pain threshold; MDT: mechanical detection threshold; MPT: mechanical pain threshold; MPS: mechanical pain sensitivity;

WUR: wind-up ratio; VDT: vibration detection threshold; PPT: pressure pain threshold. ^a^Comparison of z-score QST data between asymptomatic and symptomatic side; paired T-test; ^b^Comparison of z-score QST data measured on symptomatic side pre-and

post-surgery; paired T-test. Bold numbers indicate statistically significant difference.
