# Supplementary material for: Association of quantitative sensory testing parameters with clinical outcome in patients with lumbar radiculopathy undergoing microdiscectomy
Source: Eur J Pain. 2020 Jun 14;24(7):1377–92. doi: 10.1002/ejp.1586 (PMC7496563; doi:10.1002/ejp.1586)
Supplement: Supplementary file 3 — Table S3 [file EJP-24-1377-s003.doc]

**Supplementary Table 3**

Baseline clinical outcome measures of patients with lumbar radiculopathy who demonstrated <30% and ≥30% change on the Oswestry Disability

Index at three and at 12 months post-surgery

|  | 3 months post-surgery | | | 12 month post-surgery | | |
| --- | --- | --- | --- | --- | --- | --- |
|  | <30% change  (n = 9) | ≥30% change  (n = 44) | *p*-value | <30% change  (n = 7) | ≥30% change  (n = 41) | *p*-value |
| Symptom duration (months)* | 9.6 (5.2) | 12.1 (7.9) | .351 | 12.4 (7.0) | 11.4 (7.6) | .751 |
| Oswestry Disability Index* (0-100) | 17.2 (6.1) | 19.3 (6.2) | .645 | 15.1 (5.6) | 18.4 (6.2) | .201 |
| painDETECT* (-1-38) | 14.4 (5.0) | 16.4 (5.6) | .341 | 15.3 (4.4) | 15.8 (5.4) | .821 |
| Anxiety score (HADS)^#^ | 7.0 (7.0; 3-13) | 8.0(5.5; 2-16) | .825 | 7.0 (7.0; 2-16) | 8.0 (5.2) | .932 |
| Depression score (HADS)^#^ | 7.0 (4.0; 1-10) | 6.0 (4.5; 1-16) | .683 | 7.0 (6.0; 2-16) | 6.0(5.2) | .775 |
| Pain Catastrophizing Scale^#^ | 19.0 (14.; 1-28) | 20.0 (19.0; 1-42) | .683 | 13.0 (22.0; 1-39) | 20.5 (16.5) | .328 |
| Tampa Scale of Kinesiophobia* | 46.1 (8.2) | 44.1 (6.7) | .435 | 42.6 (9.4) | 44.5 (6.6) | .505 |
| Average leg pain last 24 hours (NRS 0-10)* | 4.7 (1.9) | 6.1 (1.9) | **.046** | 4.4 (1.9) | 6.0 (2.0) | .057 |
| Average leg pain last week (NRS 0-10)* | 4.8 (1.9) | 6.0 (1.9) | .086 | 4.3 (2.3) | 5.9 (1.8) | **.041** |
| Bothersomeness leg pain last two weeks  (0 = no at all, 5 = extremely) | 1.9 (0.8) | 2.9 (0.7) | **.000** | 2.3 (0.9) | 2.8 (0.8) | .122 |
| Average back pain last 24 hours (NRS 0-10)* | 4.1 (3.1) | 4.8 (2.3) | .446 | 4.3 (2.7) | 4.7 (2.5) | .700 |
| Average back pain last week (NRS 0-10)* | 3.7 (3.1) | 4.6 (2.4) | .313 | 3.8 (3.1) | 4.4 (2.5) | .599 |
| Bothersomeness back pain last two weeks  (0 = no at all, 5 = extremely) | 1.7 (1.1) | 2.1 (0.9) | .226 | 1.8 (0.7) | 2.1 (1.0) | .581 |
| Sleep quality during last week (VAS)^#^  (0=good sleep, 10 = bad sleep) | 3.8 (6.0; 0-10) | 5.9 (3.4; 0-10) | .400 | 4.5 (3.2; 0-10) | 5.9 (3.9) | .320 |
| SF-36 |  |  |  |  |  |  |
| Physical Component^#^ | 36.5 (5.3; 31-47) | 36.1 (8.8; 25-54) | .739 | 38.4 (11.4; 26-49) | 35.6 (7.8) | .104 |
| Mental Component^#^ | 45.5 (17.3; 32-57) | 44.2 (16.3; 17-65) | .6515 | 50.6 (16.3; 28-62) | 43.2(14.7) | .370 |

*Data are mean (SD); ^#^Data are median (IQR; minimum-maximum);

^a^Comparison baseline – 3 months post surgery; ^b^comparison baseline – 12 months post-surgery
